# Cisplatin-induced apoptosis in human malignant testicular germ cell lines depends on MEK/ERK activation

**DOI:** 10.1038/sj.bjc.6601919

**Published:** 2004-07-13

**Authors:** S Schweyer, A Soruri, O Meschter, A Heintze, F Zschunke, N Miosge, P Thelen, T Schlott, H J Radzun, A Fayyazi

**Affiliations:** 1 Department of Pathology, University of Göttingen, Robert-Koch-Str. 40, D-37075 Göttingen, Germany; 2 Department of Immunology, University of Göttingen, Robert-Koch-Str. 40, D-37075 Göttingen, Germany; 3 Department of Histology, University of Göttingen, Robert-Koch-Str. 40, D-37075 Göttingen, Germany; 4 Department of Urology, University of Göttingen, Robert-Koch-Str. 40, D-37075 Göttingen, Germany

**Keywords:** cisplatin, apoptosis, TGCT, MEK, ERK

## Abstract

Testicular germ cell tumours (TGCT) represent the most common malignancies in young males. Whereas in 1970s, the survival rate in patients with metastatic testicular tumours was only 5%, these days, 80% of the patients treated by modern chemotherapy will survive their disease. The drug that revolutionised the cure rate for patients with metastatic testicular tumours was cisdiamminedichloroplatinum (cisplatin, CDDP). *In vitro* experiments on neoplastic germ cell lines showed that their exquisite sensitivity to CDDP could be attributed to p53-dependent and -independent pathways. Applying cDNA macroarray, semiquantitative RT–PCR and Western blot analyses, blocking experiments, caspase activity assays, and morphological methods, we sought here to define the p53-independent pathway(s) involved in the CDDP-induced apoptosis. For this purpose, we used the human TGCT cell line NCCIT, the mutated p53 of which is known to remain inactive during the course of CDDP-induced apoptosis. Our experiments showed that within hours of CDDP application, two prototype members of the ‘mitogen-activated protein kinase’ (MAPK) family, designated ‘MAPK ERK kinase’ (MEK) and ‘extracellular signal-regulated kinase’ (ERK), were dually phosphorylated and caspase-3 became active. Functional assays using MEK inhibitors demonstrated that the phosphorylation of MEK and ERK was required for the activation of caspase-3 as the executing caspase. Interestingly, experiments with the human malignant germ cell line NTERA, which is known to possess wild-type p53, revealed the same results. Thus, our data suggest that CDDP mediates its p53-independent apoptosis-inducing effect on the malignant human testicular germ cells – at least partially – through activation of the MEK–ERK signalling pathway.

Except in young males, testicular germ cell tumours (TGCT) are relatively uncommon malignancies. Despite the paucity, however, TGCT have become a model for a curable neoplasm even in advanced stages. The breakthrough in the treatment of metastatic TGCT was achieved by the drug cisdiamminedichloroplatinum (cisplatin, CDDP) (Higby *et al*, 1974; Einhorn, 2002). Cisplatin is a potent inducer of apoptosis in different cell types and is one of the most effective and widely used chemotherapeutic agents in the treatment of human cancers. Although CDDP-induced apoptosis is known to be the result of its ability to damage DNA (Wyllie *et al*, 1980; Fichtinger-Schepman *et al*, 1985; Sorenson and Eastman, 1988; Eastman, 1990), the mechanisms by which CDDP initiates apoptosis in TGCT are not completely understood.

In mice, elevated levels of wild-type p53 in TGCT seem to contribute to their sensitivity to DNA-damaging drugs. Using the murine TGCT cell line F9, Lutzker and Levine (1996) demonstrated that a functionally inactive p53 protein in neoplastic germ cells became active following chemotherapy, thus leading to apoptosis. Although Chresta *et al* (1996) postulated that a hypersensitivity of human TGCT to drug-dependent apoptosis may also be associated with functional p53 , Burger *et al* (1999) showed that abrogation of the p53 function does not affect the hypersensitivity of human TGCT cell lines to chemotherapy. Thus in human TGCT, apoptotic signalling pathways including those activated by CDDP seem to be – at least partly – p53 independent (Burger *et al*, 1997; Kersemaekers *et al*, 2002).

In the present study, we sought to define the p53-independent pathway(s) involved in the CDDP-induced apoptosis of the human TGCT cell line NCCIT, the p53 of which has been shown to remain inactive during the process of CDDP-induced apoptosis (Burger *et al*, 1999). Based on results from a cDNA macroarray analysis, we assumed that CDDP exploits the ‘mitogen-activated protein kinase’ (MAPK) signalling pathway to induce apoptosis in NCCIT cells.

Mitogen-activated protein kinase constitutes a superfamily of kinases that coordinates and transduces incoming signals from stress and growth factors, hormones and cytokines (Raabe and Rapp, 2002). The most extensively studied MAPK pathway is the MAPK kinase (MEK)–extracellular signal-regulated kinase (ERK) cascade, where p-MEK1/2 phosphorylates and thereby activates ERK. Originally the ERK module was thought to mediate cell proliferation and differentiation. Recent data, however, clearly show that the ERK activation culminates in phosphorylation of many proteins with substantial regulatory functions throughout the cell, coordinating and eliciting conflicting cellular responses ranging from proliferation and differentiation to apoptosis (Peyssonnaux and Eychene, 2001; Djeu *et al*, 2002; Lee and McCubrey, 2002). Here, we show that in NCCIT cells, CDDP activates the MEK–ERK signalling pathway, which is followed by a caspase 3-mediated apoptosis.

## MATERIALS AND METHODS

### Reagents

Cisplatin was purchased from Hexal (Holzkirchen, Germany), vinblastin (vin) from Gry-Pharma (Kirchzarten, Germany) and hydrogen peroxide and two MEK1/2 inhibitors (U0126 and PD98059) from Sigma (Deisenhofen, Germany). The polyclonal antibody recognising the activated form of caspase-3 and the inhibitor of caspase-9 (Z-LEHD-FMK) were obtained from R&D systems (Wiesbaden, Germany). The horseradish-peroxidase (HRP)-conjugated polyclonal F(ab)_2_ fragment against digoxigenin was obtained from Dako (Hamburg, Germany), the monoclonal anti-actin antibody from Oncogene (Schwalbach, Germany), and inhibitors of caspase-3 (Ac-DEVD-CHO) and caspase-8 (Ac-IETD-CHO) from Pharmingen (Hamburg, Germany). The polyclonal anti-MEK1/2, anti-phospho-MEK1/2, anti-ERK1/2 and anti-phospho-ERK1/2 antibodies were purchased from Cell Signal Technology (Beverly, USA).

### Culturing and treatment of TGCT cell lines

The human TGCT cell lines used in this study were NCCIT and NTERA (American Type Culture Collection, ATCC, Manassas, USA). NCCIT and NTERA cell lines were grown as monolayers and maintained in HEPES-buffered RPMI 1640 (Biochrom, Berlin, Germany) supplemented with 10% foetal calf serum (FCS) (CC Pro, Neustadt, Germany), 100 IU ml^−1^ penicillin (Sigma, Deisenhofen, Germany), 100 *μ*g ml^−1^ streptomycin (Sigma) and 2 mM L-glutamine (Life Technologies, Karlsruhe, Germany). Cultures were incubated at 37°C in a humid atmosphere with 5% carbon dioxide in air. Then, they were plated subconfluent in 75 mm^2^ flakes and cultured in serum-free medium 1 day prior to treatment.

For the induction of apoptosis, the cells were then incubated for 2 h with different concentrations of CDDP (12.5, 25 and 50 *μ*M), vinblastin (15, 20 and 30 *μ*M) or hydrogen peroxide (300, 600 and 1200 *μ*M). After washing, cells were incubated in culture medium for further 6, 9, 12 or 24 h.

For the induction of necrosis, the cells were heated for 15, 30 or 60 min at 45°C (heat stress) or pipetted 10–15 times (mechanical stress) and were proved to be dead by reculturing.

### RNA isolation and cDNA macroarray

For cDNA macroarray, RNA was extracted 6 h after CDDP treatment. Total RNA was extracted from untreated and CDDP-treated NCCIT cells using ‘Atlas™ Pure Total RNA Labelling Kit’ (Clontech, Heidelberg, Germany). RNA integrity and quantity was assessed on an ‘Agilent Bioanalyzer 2100’ with a ‘RNA 6000 Nano LabChip Kit’ (Agilent Technologies, Waldbronn, Germany). After *DNAse* I digestion and addition of biotinylated oligo(dT), poly(A)^+^ RNA was enriched on streptavidin-labelled magnetic beads from 100 *μ*g total RNA. RNA (10 *μ*g) templates bound to the beads were subjected to cDNA synthesis with human CDS primer mixture and [*α*-^32^P]dATP. Purified cDNA probes (>6.5 × 10^6^ c.p.m.) were hybridised to the arrayed human cDNA membranes (Atlas Human Apoptosis Array, Clontech) under recommended conditions. Washed membranes were exposed against the imaging plate for 12 h at room temperature and hybridisation signals were semiquantified on the Personal Molecular Imager FX System (BioRad, München, Germany) using an image analysis software (BioRad). Gene transcripts that displayed two-fold or greater changes were considered significant.

### Reverse transcription–PCR

Total cellular RNA from pelleted cells was extracted using RNeasy Mini Kit (Qiagen, Hilden, Germany). RNA integrity and quantity was assessed on an Agilent Bioanalyser 2100 with a RNA 6000 Nano LabChip®-Kit (Agilent Technologies, Waldbronn, Germany). Reverse transcription of 500 ng total cellular RNA with random hexamer primers was performed with Omniscript RT Kit (Qiagen). Expression of MEK1 and MEK2 was quantitated on an iCycler iQ real-time detection system (BIORAD, Munich, Germany) with HotStar *Taq* DNA Polymerase Kit (Qiagen). The 20 *μ*l reaction from the kit was supplemented with 2 *μ*l cDNA, 0.6 *μ*M gene-specific primers and SYBR green (MoBiTec, Göttingen, Germany). Primers (MWG, Ebersberg, Germany) were designed using primer3 on-line primer design program (www-genome.wi.mit.edu/cgi-bin/ primer/primer3_www.cgi).

MEK1 forward primer: 5′-TGAGAAGATCAGTGAGCTGG-3′; MEK1 reverse primer: 5′-ACTTGATCCAGAGAACCTCC-3′; MEK2 forward primer: 5′-AACTCAAAGACGATGACTTCG-3′; MEK2 reverse primer: 5′-CCATGCAAATGCTGATCTCC-3′. Acquisition of fluorescence signals was monitored on the iCycler and terminated, when all reactions reached an amplification plateau while a template-free control remained at basement level. Data analysis was carried out with iCycler iQ real-time detection system software (BioRad). To verify that only specific PCR products evoked fluorescence signals, PCR products were analysed by melt curve (BioRad iCycler) and run on 2% agarose gels for densitometry with E.A.S.Y. Win 32 software (HEROLAB, Wiesloch, Germany).

### Morphological detection, identification and quantification of apoptosis

#### May–Giemsa–Grunwald staining

Centrifuged cells (2 × 10^3^) were dried for 24 h, fixed in 100% acetone for 10 min, stained with May–Giemsa–Grunwald and embedded in ‘SuperMount Medium’ (Dako). Apoptotic cells were identified by cellular shrinkage, nuclear condensation and fragmentation.

#### *In situ* end labelling (ISEL)

Fixed centrifuged cells (2 × 10^3^) were rinsed with TBS (50 mM Tris-HCl; 150 mM NaCl; pH 7,5) containing 10% FCS and 0.3% H_2_O_2_ to block endogenous peroxidase activity. The cells were then incubated for 60 min at 37°C with 50 *μ*l of the labelling mix (250 U × ml^−1^ terminal transferase, 20 *μ*L ml^−1^ digoxigen–DNA labelling mix at 10 × concentration, and 1 mmol l^−1^ CoCl_2_ in reaction buffer for terminal transferase (Roche, Mannheim, Germany)). After being rinsing in TBS, the cells were blocked with 10% FCS and incubated for 60 min with a rabbit HRP-conjugated F(ab)_2_ fragment against digoxigenin (working dilution of 1 : 500; Dako). 3,3′-Diaminobenzidine (DAB) was next applied as chromogen. Cells with fragmented DNA revealed nuclear brown signals. DNA-fragmented cells with intact plasma membrane were considered to be apoptotic. Negative controls were stained as above but without terminal transferase.

#### Immunocytochemistry

Fixed centrifuged cells (2 × 10^3^) were rinsed in TBS containing 10% FCS and 0.3% H_2_O_2_ for 10 min. Subsequently, cells were incubated with the polyclonal antibody against active caspase-3 for 30 min. After washing in TBS, cells were further incubated with a biotin-conjugated polyclonal goat anti-rabbit antibody (Dako) for another 20 min. The cells were then incubated with HRP-conjugated streptavidin (Dako) for 20 min. The signals were visualised with DAB. Positive cells showed a brown cytoplasmic staining around the clearly demarcated nuclei. Controls were stained as above omitting primary and secondary antibody.

#### Identification and quantification of apoptosis

Apoptotic cells were identified by nuclear condensation and fragmentation in MGG staining, by positive cytoplasmic signals for the active form of caspase-3 in immunocytochemistry or by DNA fragmentation in ISEL. Percentage of apoptotic cells was calculated as the ratio of apoptotic cells to 500 cells counted. All experiments were repeated three times with similar results.

### Fluorogenic substrate assay for caspase activity

Caspase-3, caspase-8 and caspase-9 activities were examined using commercially available fluoremetric kits (caspase-3 and -8 assays, Santa Cruz Biotechnology, Heidelberg, Germany; caspase-9 assay, BioRad). Following harvesting and centrifugation, cells (1 × 10^5^) were lysed in 100 *μ*l lysis buffer and incubated for 10 min in 100 *μ*l of the reaction buffer supplemented with 100 *μ*M of the fluorogenic peptide substrate Ac-DEVD-AMC, Ac-IETD-AMC, or Ac-LEHD-AMC to measure caspase-3, -8, or -9 activity, respectively. When necessary caspase activity was inhibited by Ac-DEVD-CHO (specific inhibitor of caspase 3), or by Ac-IETD-CHO (specific inhibitor of caspase-8) or by Z-LEHD-FMK (specific inhibitor of caspase-9) as recommended by the manufacturer. After 1 h, the release of fluorescent 7-amino-4-trifluoromethyl coumarin was measured at 5-min intervals on a ‘Fluoroskan Ascent’ (Labsystems, Helsinki, Finland). All experiments were performed in triplicate and repeated three times. The data were expressed as the increase of fluorescence as a function of time.

### Western blot

Tumour cells were harvested at 10^7^ cells in 500 *μ*l of lysis puffer (0.4% sodium deoxycholate, 1% NP-40, 50 mM EGTA, pH 7.4, 10 mM Tris pH 7.4, 1 mM phenylsulphonyl fluoride (PMSF), 10 *μ*g ml^−1^ leupeptin, 10 *μ*g ml^−1^ aprotinin). After standing on ice for 20 min, the cell lysates were cleared by centrifugation at 5000 *g* for 10 min. The cytosolic supernatants containing equal amounts of protein were then resolved by electrophoresis on SDS–PAGE gel (10% (w v^−1^) gel). Thereafter, the proteins were transferred electrophoretically to a nitrocellulose membrane (Schuett, Goettingen, Germany). After blocking in 10 mM Tris-HCl buffer, pH 7.4, containing 150 mM NaCl, 0.1% Tween 20 and 5% (w v^−1^) nonfat dry milk, the membrane was treated with appropriate primary antibodies followed by incubation with HRP-conjugated secondary antibodies. The antigen–antibody complexes were detected using a chemiluminescence reagent kit (Amersham Pharmacia Biotech, Freiburg, Germany).

### Cell cycle analysis

To exclude that MEK inhibitors impair the cell cycle, NCCIT cells were stained by CycleTEST™ Plus DNA Reagent Kit (Becton Dickinson, Heidelberg, Germany) and analysed by flow cytometry (FACSCalibur, Becton Dickinson), as described by others (Bach *et al*, 1991).

## RESULTS

### Dose- and time-dependent effects of CDDP

**Figure 1:**
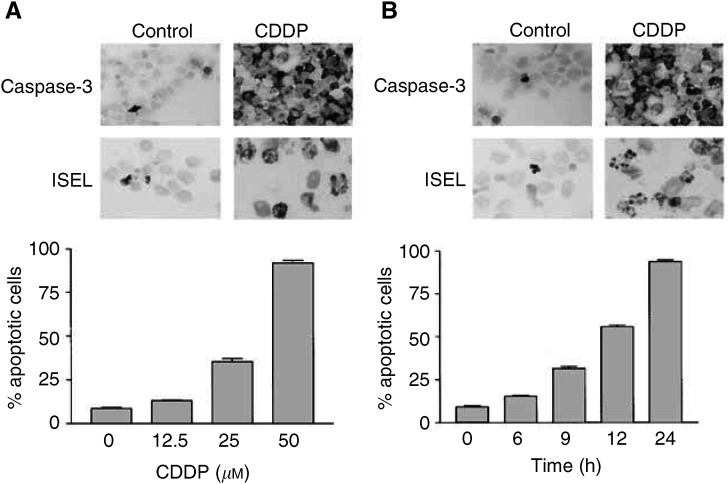
Dose- and time-dependent effects of CDDP on NCCIT cells. (**A**) Tumour cells were treated with different CDDP concentrations. After 24 h, the apoptotic rate of tumour cells was assessed by MGG staining, immunocytochemistry for the active form of caspase-3 and ISEL for DNA fragmentation. Representative photomicrographs of active caspase-3 (black cytoplasmatic signals) and of DNA fragmentation (black nuclear signals) in untreated (control) and drug-treated NCCIT cells illustrate increased apoptosis of tumour cells treated with 50 *μ*M CDDP when compared to the control. The diagram shows quantification of tumour cell apoptosis with MGG staining following application of different CDDP concentrations. (**B**) Tumour cells were treated with 50 *μ*M CDDP. After 6, 9, 12 and 24 h, the apoptotic rate of tumour cells was assessed. Representative photomicrographs of active caspase-3 and of DNA fragmentation in untreated and CDDP-treated NCCIT cells illustrate increased apoptosis of tumour cells 24 h after drug treatment. The diagram shows quantification of tumour cell apoptosis with MGG staining in time dependency. Data presented in (**A**) and (**B**) are the mean of triplicates; similar results were obtained in three separate experiments.

Dose-dependent effects of CDDP were determined 24 h after application of 12.5, 25 or 50 *μ*M CDDP to the NCCIT cells. To assess the apoptotic rate in addition to MGG, we applied two other morphological methods, namely ISEL for DNA fragmentation and immunocytochemistry for the active form of caspase 3. A concentration of 50 *μ*M CDDP was defined as the optimal drug concentration to elicit apoptosis in 93±5% of tumour cells within 24 h (Figure 1

). Experiments with this optimal CDDP concentration revealed that the apoptotic rate of NCCIT cells increased from 9±2% at the beginning to 15±1% at 6 h, 31±2% at 9 h, 54±4% at 12 h and 93±5% at 24 h after drug application (Figure 1).

### Analysis of apoptosis-regulating genes following CDDP treatment

On the strength of the evidence that the apoptotic rate of tumour cells showed an initial increase at 6 h after drug treatment, we performed a cDNA macroarray analysis to compare the expression pattern of 205 apoptosis-related genes in NCCIT cells before and 6 h after application of 50 *μ*M CDDP. Most of the genes analysed, however, were either not detectable (no signal) or only weakly expressed in NCCIT cells (weak signals) and did not reveal any down- or upregulation after drug treatment. p53, the p53-regulated gene mdm-2 and genes from the Bcl-2 family belonged to this group, as reported by others (Burger *et al*, 1997; Kersemaekers *et al*, 2002) (Table 1

**Table 1:** Expression of apoptosis-related genes in NCCIT cells following CDDP treatment

CDK4	Cyclin-dependent kinase 2	Increased
CDK5	Cyclin-dependent kinase 4	Increased
Cyclin B1	Cyclin B	Increased
Caspase-3	Caspase-3	Increased
GRB-2	Growth factor receptor-bound protein 2	Increased
PLK	Polo-like kinase	Increased
p53	p53 tumor suppressor protein	Unchanged
mdm-2	p53-binding mouse double minute 2 homolog	Unchanged
bcl-2	B-cell leukemia/lymphoma protein 2	Unchanged
bax	Bcl-2-associated X protein membrane	Unchanged
IGFBP-2	IGF-binding protein 2	Decreased
AP-1	Transcription factor	Decreased
CDC25A	Cell division cycle 25 homolog A	Decreased
GADD45	Growth arrest and DNA damage-inducible protein 45	Decreased
ID-1	Inhibitor of DNA-binding protein 1	Decreased
MEK1/2	Mitogen-activated protein kinase kinase 1/2	Sustained high
RHOA	Ras homolog gene family member	Sustained high
Prefoldin 5	c-myc-binding protein	Sustained high
N-myc	N-myc	Sustained high

Two RNA preparations were isolated from untreated and CDDP-treated NCCIT cells. RNAs were reverse transcribed, and generated cDNA probes were hybridised to cDNA macroarray membrane filters. Gene transcripts that displayed two-fold or greater changes were considered significant. Only 17 differentially expressed genes were identified among 205 genes investigated.

). The expression level of five genes including IGFBP-2, AP-1, CDC25A, GADD45 and ID-1 was decreased in treated cells compared to the levels in untreated cells (Table 1). In contrast, the expression of 12 genes including those involved in cell cycle and apoptosis (e.g. CDK4, CDK5, cyclin B1 and caspase-3), or in signal transductions (e.g. GRB2 and PLK1) was increased in CDDP-treated cells when compared to untreated ones (Table 1). The fourth category of genes included those with a sustained high level of expression before and after drug treatment. MEK1/2, RHOA, Prefoldin and N-myc belonged to this category of genes (Table 1).

**Figure 2:**
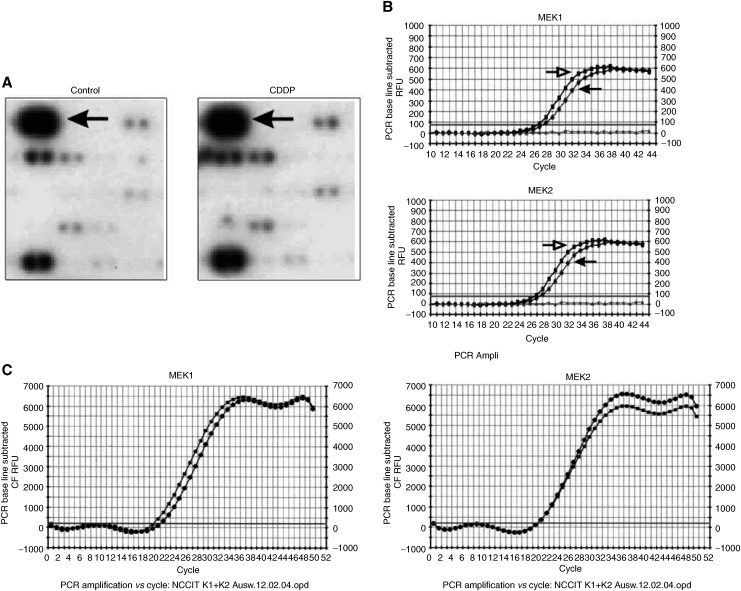
MEK1/2 mRNA expression in untreated and CDDP-treated NCCIT cells. (**A**) Two RNA preparations from untreated and CDDP-treated NCCIT cells were reverse transcribed and hybridised to cDNA macroarray membrane filters. Sections from two representative autoradiographies show sustained expression of MEK1/2 in untreated (control) and CDDP-treated (50 *μ*M) NCCIT cells 6 h after drug treatment (arrows). (**B**) semiquantitative real-time RT–PCR of MEK1 and MEK2 mRNA confirms that the expression level of both transcripts remains constant in CDDP-treated NCCIT cells (open arrows, left) compared to untreated ones (closed arrows, right). (**C**) as control, the expression level of MEK1 and MEK2 mRNA after heat-induced necrosis of the NCCIT cell line was analysed. Results demonstrate that in the course of heat-induced necrosis, the expression level of MEK mRNAs is not changed (-•-) compared to untreated ones (-▪-).

Owing to the high and sustained expression level of MEK1/2 mRNA, and the fact that activation of the MEK–ERK pathway cannot only hamper but also promote the sensitivity of tumour cells to CDDP (Persons *et al*, 1999; Wang *et al*, 2000), in the follow-up this study was focused on the role of MEK in the course of drug-induced apoptosis. At first, we proved the expression of MEK1/2 mRNA by a second method. Semiquantitative RT–PCR confirmed the results from the macroarray and revealed that in NCCIT cells the transcription level of both MEK1 and MEK2 mRNAs remained constant after drug treatment (Table 1, Figure 2

). As control, we investigated the expression level of MEK1/2 mRNA after heat- and mechanically induced necrosis of the NCCIT cell line. Results showed that stress stimuli leading to cell death without primary DNA damage (heat- or mechanically induced necrosis) do not elicit any significant change in the expression level of MEK1/2 mRNA. Figure 2 illustrates the expression level of MEK1 and MEK2 mRNAs following heat-induced necrosis in NCCIT cells.

### CDDP-induced activation of the MEK–ERK signalling pathway

**Figure 3:**
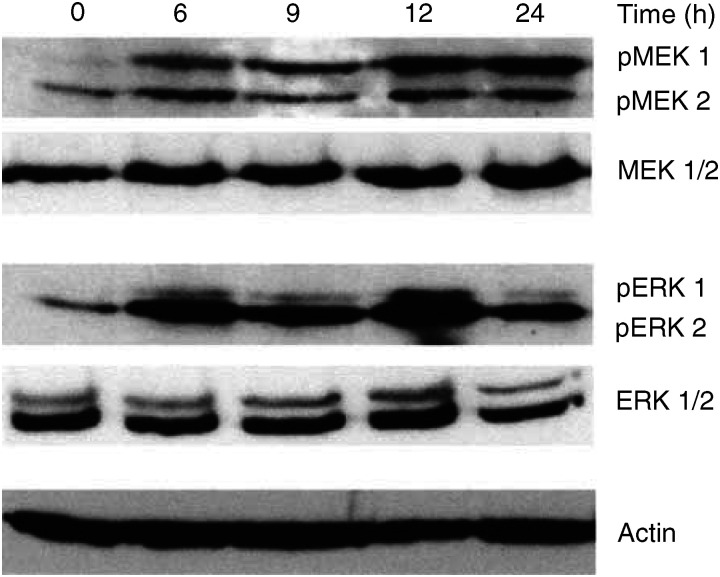
CDDP-induced activation of the MEK/ERK pathway in NCCIT cells. NCCIT cells were treated with 50 *μ*M CDDP for 2 h, after which cell lysates were investigated for the expression and activation (phosphorylation) of MEK1/2 and ERK1/2 by Western blot analysis in the time course. Upper panel illustrates the expression of MEK1/2 and its activation (phosphorylation) following CDDP treatment. Lower panel demonstrates results from Western blots of ERK1/2 and its activated (phosphorylated) form pERK1/2 in the time course after CDDP treatment. Note that MEK1 and ERK2 are more strongly phosphorylated than MEK2 and ERK1. Expression of actin was used to control equal protein loading.

Next, lysates from untreated and drug-treated tumour cells were analysed by Western blot for the expression of MEK1/2 and its substrate ERK1/2. The same blots were stripped and analysed with antibodies recognising dually phosphorylated MEK (pMEK) 1/2 or dually phosphorylated ERK (pERK) 1/2 to verify the active fraction of each protein in our experiments. Results illustrated that, despite high expression, MEK1/2 and ERK1/2 proteins were mainly unphosphorylated in untreated NCCIT cells (Figure 3

). However, phosphorylation of MEK1/2 and ERK1/2 proteins began to increase at 6 h and was at its highest at 12 h after CDDP treatment, a course which has been observed in growth factor- or oxidant-mediated MEK–ERK activation in many cell types (Jimenez *et al*, 1997; Wang *et al*, 1998). Interestingly, MEK1 and ERK2 seem to be more strongly phosphorylated than MEK2 and ERK1.

### Blocking of CDDP-induced apoptosis by MEK inhibitors

**Figure 4:**
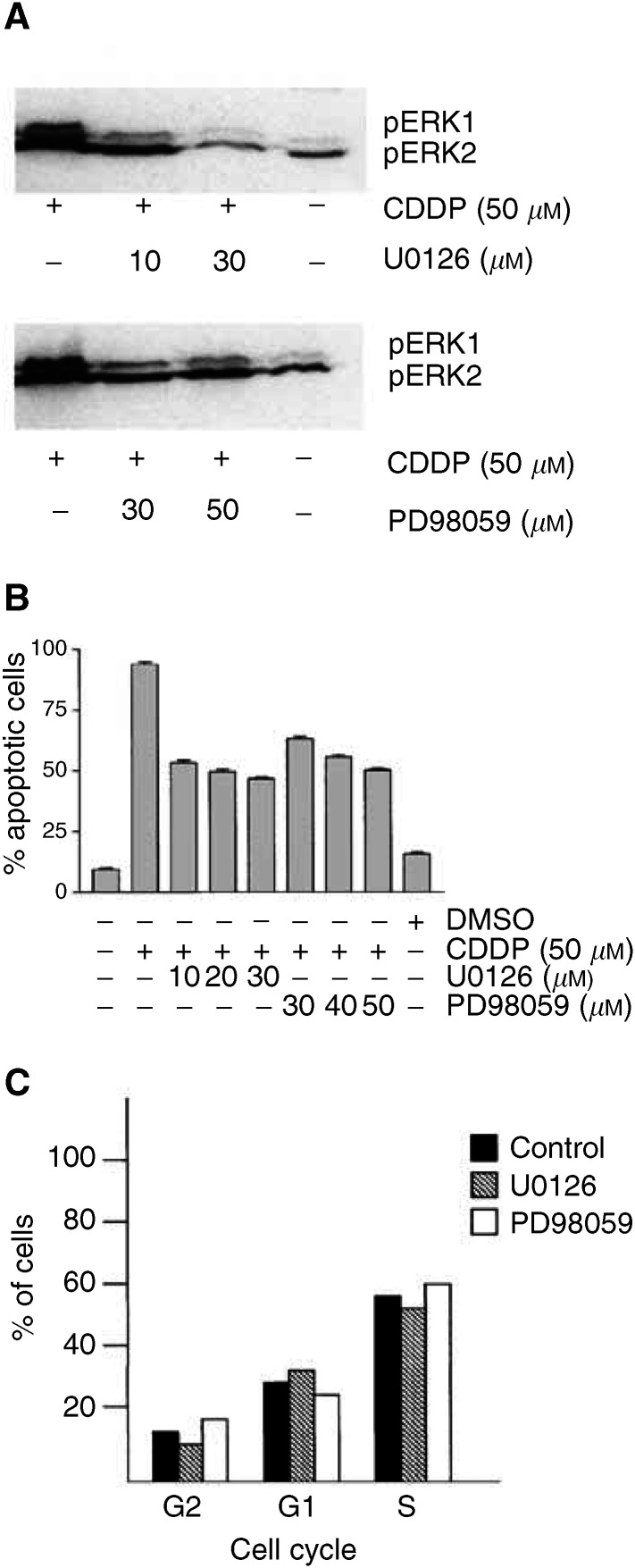
Blocking of CDDP-induced apoptosis by MEK inhibitors. To investigate whether phosphorylation of ERK1/2 is required for the CDDP-induced apoptosis of NCCIT cells, two different MEK inhibitors, U0126 and PD98059, were applied in addition to 50 *μ*M CDDP. (**A**) Western blot analysis with an anti-phospho-ERK1/2 antibody shows the inhibition of ERK1/2 activation by MEK1/2 inhibitors (U0126, 10 and 30 *μ*M; PD98059, 30 and 50 *μ*M). (**B**) The diagram shows quantification of tumour cell apoptosis with MGG staining dependent on ERK blockage by different concentration of MEK inhibitors after 24 h. Data are the mean of triplicates; similar results were obtained in three separate experiments. (**C**) Flow cytometric DNA analysis of NCCIT cells treated with the MEK inhibitor U0126 (50 *μ*M) demonstrates that following drug treatment, the cell cycle of NCCIT cells is not changed.

To study whether ERK activation is required for CDDP-induced apoptosis in NCCIT cells, two chemical MEK1/2 inhibitors, U0126 and PD98059, were used. Cells were treated with various doses of MEK1/2 inhibitors simultaneously to the addition of 50 *μ*M CDDP. Both inhibitors reduced the grade of ERK phosphorylation (Figure 4

). Morphological quantification of apoptotic cells by MGG, immunostaining for active caspase 3 and ISEL for fragmented DNA demonstrated that application of an inhibitor significantly reduced the CDDP-induced apoptosis of tumour cells in a dose-dependent manner when compared to controls. For example, application of 30 *μ*M U0126 was capable of decreasing the rate of apoptosis of drug-treated cells from approximately 95 to about 50%. Results are shown in Figure 4. To rule out that MEK inhibitors unspecifically influence the cell cycle and thereby interfere with apoptosis, flow cytometric DNA analysis of NCCIT cells were carried out 6 and 24 h after treatment with U0126 or PD98059 (50 *μ*M). Results showed that MEK inhibitors do not impair the cell cycle of NCCIT cells (Figure 4).

### MEK–ERK activation results in caspase activity

**Figure 5:**
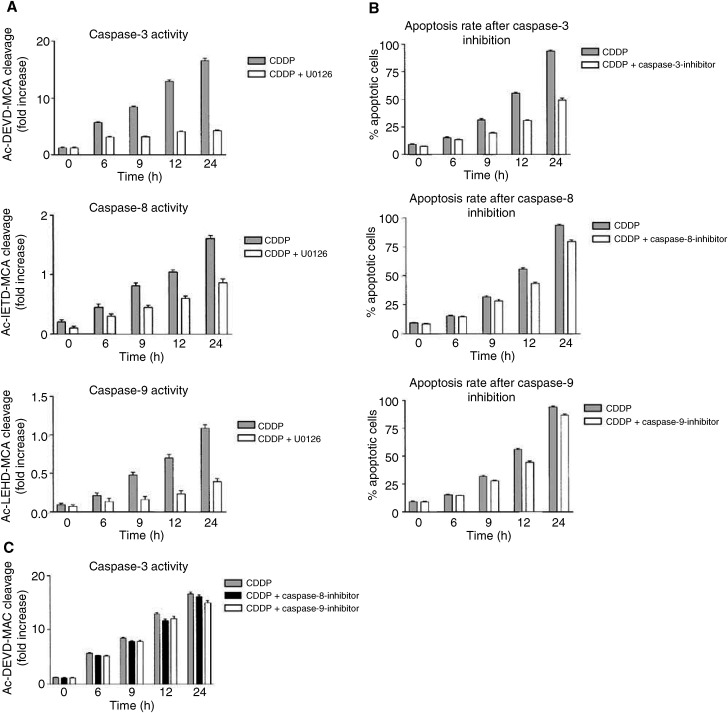
MEK–ERK activation results in caspase activity. (**A**) NCCIT cells were treated with CDDP (50 *μ*M) in the presence or absence of the MEK inhibitor U0126 (30 *μ*M) as described in Materials and methods. After cell lysis, the activity of caspase-3, -8 and -9 was determined using specific fluorogenic substrates: Ac-DEVD-MCA (for caspase-3), Ac-IETD-MCA (for caspase-8) and Ac-LEHD-MCA (for caspase-9). The data are expressed as the increase in fluorescence as a function of time. Note that the *Y*-axis ranges from 0 to 20 for caspase-3, from 0 to 2 for caspase-8 and from 0 to 1.5 for caspase-9. (**B**) NCCIT cells were treated with CDDP (50 *μ*M) in the presence or absence of Ac-DEVD-CHO as caspase-3 inhibitor, or Ac-IETD-CHO as caspase-8 inhibitor or Z-LEHD-FMK as caspase-9 inhibitor. Diagrams show quantification of tumour cell apoptosis with MGG staining in time dependency. (**C**) NCCIT cells were treated with CDDP (50 *μ*M) in the presence or absence of Ac-IETD-CHO as caspase-8 inhibitor or Z-LEHD-FMK as caspase-9 inhibitor. After cell lysis, the activity of caspase-3 was determined. Results show the increase in fluorescence as a function of time. Data presented in (**A–C**) are the mean of triplicates; similar results were obtained in three separate experiments.

By applying caspase assays, we first analysed the activation of initiating caspase-8 and -9 and of the executing caspase-3 in the course of CDDP-induced apoptosis in NCCIT cells. Results demonstrated that the caspase-8 and -9 activity increased 3–4-fold, and the activity of caspase-3 about 14–16-fold at 24 h after CDDP application (Figure 5

). Next we investigated the activity of caspase-8, -9 and -3 following MEK inhibition to ascertain that the increased activation of these caspases depended on the MEK–ERK activation. Indeed, application of MEK inhibitors (U0126 or PD98059) substantially decreased the activity of all three caspases (Figure 5). We studied then the biological influence of each caspase for apoptosis on drug-treated NCCIT cells. We noted that 24 h after CDDP application, the apoptotic rate of tumour cells decreased from 93±5 to 75±4% following inhibition of caspase-8, to 85±3% following inhibition of caspase-9, or to 49±6% following inhibition of caspase-3 (Figure 5). Moreover, we observed that the activity of caspase-3 was only weakly reduced when caspase-8 or -9 was inhibited (Figure 5). Thus, caspase-8 and -9 seem to play only a supporting role, whereas caspase-3 is the main caspase responsible for the CDDP-induced apoptosis in NCCIT cells.

### Selectivity of CDDP-induced apoptosis

**Figure 6:**
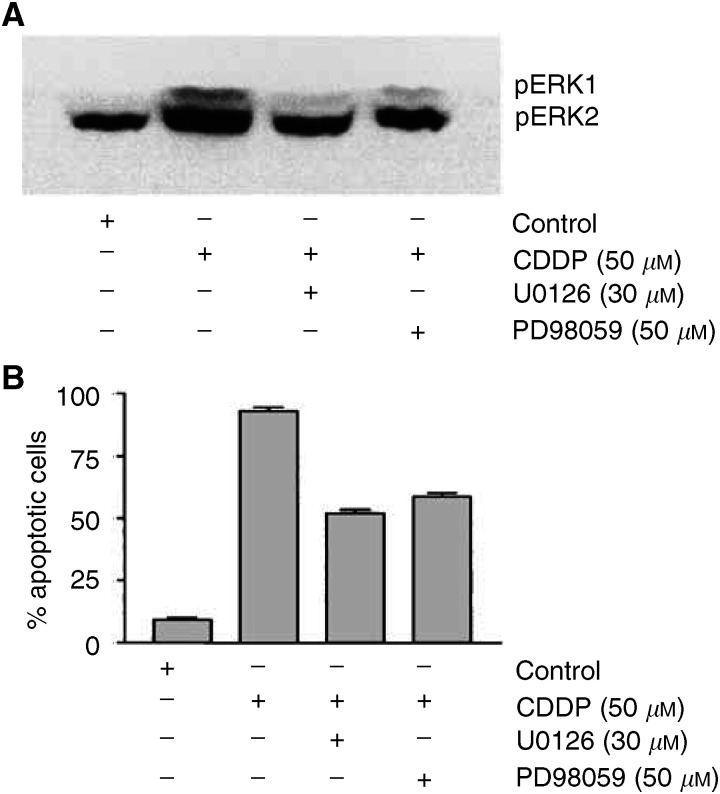
Selectivity of CDDP-induced apoptosis. NTERA cells were treated with 50 *μ*M CDDP in the presence or absence of the MEK inhibitors U0126 (30 *μ*M) or PD98059 (50 *μ*M), as described in Material and methods. (**A**) After 24 h, tumour cells were lysed and investigated for the expression and activation (phosphorylation) of ERK1/2 by Western blot analysis. (**B**) the diagram shows quantification of tumour cell apoptosis with MGG staining dependent on ERK blockage by MEK inhibitors after 24 h. Data are the mean of triplicates; similar results were obtained in three separate experiments.

To prove whether activation of the MEK/ERK pathway seen in the p53-mutated TGCT cell line NCCIT is also required for the CDDP-induced apoptosis in human neoplastic germ cells with wt-p53, NTERA cells were treated with CDDP. As reported by others (Chresta *et al*, 1996; Burger *et al*, 1999), CDDP treatment led to apoptosis of NTERA cells dose and time dependently (data not shown). Western analysis showed that following CDDP application (50 *μ*M) both MEK and ERK were phosphorylated and simultaneous treatment of NTERA cells with CDDP and one of the MEK1/2 inhibitors U0126 (30 *μ*M) or PD98059 (50 *μ*M) significantly reduced the apoptosis. Notably, the effect of MEK1/2 inhibitors on the apoptotic rate of drug-treated NTERA cells was as efficient as that seen in NCCIT cells (Figure 6

). Thus, we concluded that the CDDP-induced apoptosis of NCCIT and NTERA cells was, at least partially, p53 independent and mediated by the MEK/ERK signalling pathway.

### Specificity of CDDP-induced apoptosis

**Figure 7:**
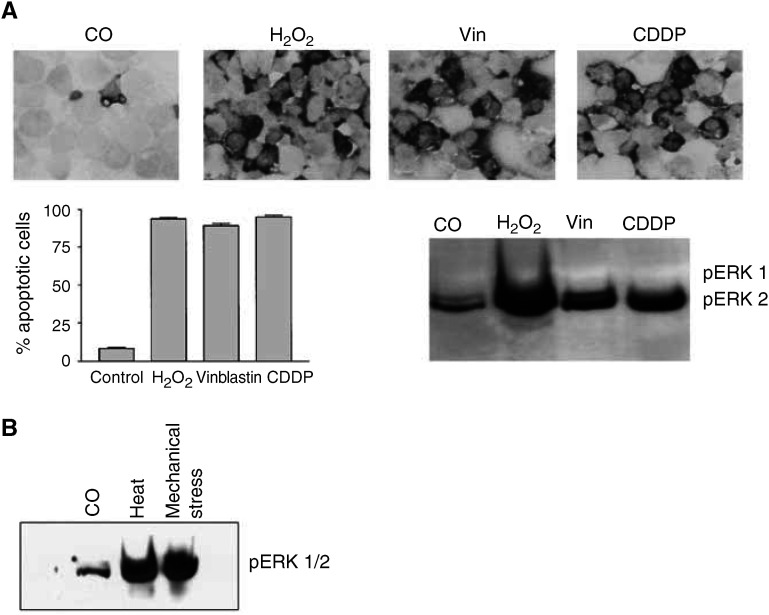
Specificity of CDDP-induced apoptosis. (**A**) NCCIT cells were treated with CDDP (50 *μ*M), vinblastin (30 *μ*M) or H_2_O_2_ (1200 *μ*M). After 24 h, the apoptotic rate of tumour cells was assessed by immunocytochemistry for the active form of caspase-3 and MGG staining. Representative photomicrographs of caspase-3 (black cytoplasmatic signals) in NCCIT cells illustrate increased caspase activity in tumour cells following treatment. The diagram shows quantification of tumour cell apoptosis with MGG staining. Data are the mean of triplicates; similar results were obtained in three separate experiments. A fraction of tumour cells was lysed and investigated for the expression and activation (phosphorylation) of ERK1/2 by Western blot analysis. (**B**) To examine the role of the MEK–ERK signalling pathway in the course of necrosis, NCCIT cells underwent heat (45°C up to 60 min) or mechanical stress (multiple pipetting). Results demonstrate that stress stimuli leading to cell death without primary DNA damage (heat- or mechanically induced necrosis) also elicit ERK phosphorylation.

To examine the role of the MEK–ERK signalling pathway in the induction of apoptosis and/or necrosis following other stress stimuli, NCCIT cells were treated with various doses of vinblastin (15–30 *μ*M) – as a relevant drug in the therapy of TGCT – and of hydrogen peroxide (H_2_O_2_; 300–1200 *μ*M) or underwent heat (45°C up to 60 min) or mechanical stress (multiple pipetting). Figure 7

demonstrates that treatment with vinblastin and H_2_O_2_ led to apoptosis of NCCIT cells in a dose-dependent manner and was conducted by ERK activation. As expected, MEK inhibition led to a significant reduction in apoptosis in NCCIT cells and was as effective as that seen in experiments with CDDP and MEK1/2 inhibitors U0126 or PD98059 (data not shown). Results also show that stress stimuli leading to cell death without primary DNA damage (heat- or mechanically induced necrosis) elicit ERK phosphorylation (Figure 7).

## DISCUSSION

The present study was focused on p53-independent mechanisms involved in CDDP-induced apoptosis of the human neoplastic germ cell line NCCIT, the p53 protein of which is known to remain inactive during the course of drug-induced apoptosis (Burger *et al*, 1997; Burger *et al*, 1999). To address this issue, we first studied dose- and time-dependent effects of CDDP on NCCIT cells. Experiments indicated that following application of an optimal concentration of 50 *μ*M CDDP, the apoptotic rate of tumour cells increased from approximately 10% at the beginning to about 95% at 24 h. Significant differences in the degree of apoptosis between CDDP-treated and untreated cells were, however, not observed before 6 h. In the light of previous data indicating that CDDP exerts its cytotoxicity via the formation of mono-, inter-, and intrastrand CDDP-DNA adducts, which can ultimately result in cell cycle arrest at G1, S or G2-M and in the induction of apoptosis (Fichtinger-Schepman *et al*, 1985; Sorenson and Eastman, 1988; Eastman, 1990), we hypothesised that within 6 h after application CDDP upregulated proapoptotic and/or downregulated antiapoptotic genes, thus mediating apoptosis in NCCIT cells. For a scanning view of differentially regulated genes, we investigated the expression profile of 205 apoptosis-related genes by applying a commercially available cDNA macroarray. As expected, no significant changes were found in the expression levels of p53 and the p53-dependent gene mdm-2 upon drug application. In addition to a few differentially regulated genes with unknown function for the course of CDDP-induced apoptosis, we noted a sustained high level of MEK1/2 mRNA expression in NCCIT cells before and after drug treatment.

MEK1 and MEK2 are protein kinases that when activated (phosphorylated) are believed to dually phosphorylate only ERK1 and ERK2, thereby increasing the enzymatic activity of ERKs approximately 1000-fold over the activity found with the basal or monophosphorylated forms (Traverse *et al*, 1992; Lenormand *et al*, 1993, Robbins *et al*, 1993). The consequence is a phosphorylation of diverse protein kinases, transcription factors, and even cytoskeletal proteins leading to paradoxical cellular responses ranging from proliferation to apoptosis (Peyssonnaux and Eychene, 2001; Djeu *et al*, 2002; Lee and McCubrey, 2002). Interestingly, similar conflicting reports also exist on the role of the MEKs and ERKs in CDDP-induced apoptosis. For example, the activity of the MEK–ERK signalling pathway seems to reduce sensitivity to CDDP in ovarian carcinoma cells, whereas phosphorylation of MEK and ERK has been found to be essential for the CDDP-induced apoptosis in cervical carcinoma cells (Persons *et al*, 1999; Wang *et al*, 2000).

To prove the results from the cDNA macroarray analysis and to address the role of the MEK–ERK signalling pathway in neoplastic germ cells, semiquantitative RT–PCR and immunoblots were carried out. The findings from the real-time RT–PCR were in agreement with those from the cDNA macroarray analysis and indicated that up to 24 h after CDDP application the expression level of MEK1 and MEK2 mRNAs remained constant. Immunoblots supported these data and demonstrated that both MEK1/2 and ERK1/2 proteins were strongly expressed but were mainly unphosphorylated and thereby inactive in untreated tumour cells. Interestingly, CDDP treatment resulted in high and sustained activation of MEK, particularly MEK1, and ERK, especially ERK2, which was strongly correlated to the apoptosis of tumour cells. Thus, it is tempting to speculate that a prolonged period of excessive phosphorylation of ERK may be necessary for its biological effect on TGCT as demonstrated for other cells types (Traverse *et al*, 1992; Lenormand *et al*, 1993).

To clarify the importance of the MEK–ERK pathway for the apoptosis of drug-treated NCCIT cells, we interrupted the pathway activity by using two different MEK inhibitors. Experiments revealed that the MEK inhibitors not only downregulated the level of ERK phosphorylation but also reduced the apoptotic rate of CDDP-treated NCCIT cells, as previously reported for the cervical carcinoma cell line HeLa (Wang *et al*, 2000). Results from RT–PCR and Western analyses, however, suggested that the CDDP-induced apoptosis in NCCIT cells depended on phosphorylation of MEK and ERK constitutively expressed in tumour cells and did not require any MEK/ERK *de novo* expression.

Considering the pivotal role of caspases in the initiation and execution of apoptosis (Thornberry and Lazebnik, 1998), we next analysed the activation of caspase-3, -8 and -9 following CDDP treatment of NCCIT cells. Caspases are divided into two subfamilies, initiator (e.g. caspase-8) and executor (e.g. caspase-3), based on their roles in the apoptotic signalling cascade (Salvesen and Dixit, 1997; Nunez *et al*, 1998). Depending on apoptotic signal and cell type, activation of initiator caspases may lead to mitochondria-dependent and/or -independent activation of executor caspases; whereas the former requires cytochrome *c* release and activation of caspase-9 as consequences of mitochondria damage, the latter is characterised by a cytochrome-*c-* and caspase-9-independent activation of executor caspases (Krammer, 2000). In this study, elevated caspase activity after CDDP application was found in caspase-3, -8 and -9. However, caspase-3 activation seemed to be more important for the course of apoptosis than caspase-8 and -9 activation, because blockade of caspase-3 inhibited apoptosis to a greater extent than the blockade of caspase-8 or -9. Consistently, the activity of caspase-3 was only moderately abolished when caspase-8 or -9 was blocked. Taking the published data on the involvement of caspase-8 and -9 in mitochondria-dependent apoptosis into account (Krammer, 2000), we consider the possibility that ERK-mediated apoptosis in NCCIT cells is mainly induced by a mitochondria-independent activation of caspase-3.

We investigated then whether the ERK-mediated apoptosis is specific for NCCIT, and whether other apoptosis-inducing agents also activate ERK in NCCIT cells. Experiments on the human malignant germ cell line NTERA possessing the wild-type p53 had indicated that NTERA cells were as sensitive to CDDP as NCCIT cells, and apoptosis of NTERA cells was also mediated by ERK. Consequently, when MEK was blocked by specific inhibitors, the apoptotic rate of treated NTERA cells was reduced significantly. Thus, activation of the MEK–ERK pathway leads to apoptosis independent from the p53 status. Importantly, experiments with another relevant chemotherapeutic drug (vinblastin) and reactive oxygen species (H_2_O_2_) revealed that following treatment of neoplastic germ cells ERK was phosphorylated, and tumour cells underwent apoptosis. Thus, activation of the MEK–ERK pathway enhances apoptosis not only in NCCIT but also in NTERA cells and the ERK-mediated apoptosis in NCCIT cells is not limited to CDDP. To answer the question whether ERK activation can also be induced by nonapoptotic stimuli inducing cell death without primary DNA damage, NCCIT cells underwent heat or mechanical stress. Results showed that these stress stimuli also led to the ERK phosphorylation. In conclusion, phosphorylation of ERK is not specific for CDDP, because other apoptosis (e.g. Vinblastin)- and necrosis-inducing stimuli (e.g. heat) are also capable to activate the ERK signalling pathway.

The question is, however, how does the MEK–ERK pathway becomes active following treatment with drugs (e.g. CDDP or vinblastin) or reactive oxygen species (e.g. H_2_O_2_). In this respect, recent data support the hypothesis that toxic substances such as reactive oxygen species may inhibit phosphatases (e.g. protein phosphatase or protein tyrosine phosphatases) and thereby contribute to the activation of ERK (Lee and Esselman, 2002) Another scenario may be that CDDP ligates growth factor receptors, thereby activating the MEK–ERK pathway, as suggested for the cervical carcinoma cell line HeLa (Wang *et al*, 2000). Indeed there are even some reports demonstrating that TGCT express an aberrant platelet-derived growth factor *α*-receptor, which is hypothetically capable of activating the MEK–ERK pathway upon ligation (Kollmannsberger *et al*, 2002; Palumbo *et al*, 2002).

Other remaining questions are whether other kinases (e.g. JNK or p38) collaborate with MEK–ERK in apoptosis of malignant germ cells, as shown for some tumours (Boldt *et al*, 2002), and – more importantly – which downstream effectors are involved in MEK–ERK-mediated apoptosis?

In conclusion, the data suggest that CDDP mediates its p53-independent apoptosis-inducing effect on the malignant human testicular germ cells through activation of the MEK–ERK signalling pathway that culminates in activation of the executor caspase-3 and thereby in programmed cell death. The data would also suggest that an antitumour therapy with CDDP should not be combined with MEK inhibitors because such combination might be antagonistic and not of benefit for the outcome of patients with TGCT.
